# Evaluation of the Enzymatic Arsenal Secreted by *Myceliophthora thermophila* During Growth on Sugarcane Bagasse With a Focus on LPMOs

**DOI:** 10.3389/fbioe.2020.01028

**Published:** 2020-08-25

**Authors:** Maria Angela B. Grieco, Mireille Haon, Sacha Grisel, Ana Lucia de Oliveira-Carvalho, Augusto Vieira Magalhães, Russolina B. Zingali, Nei Pereira, Jean-Guy Berrin

**Affiliations:** ^1^Laboratório de Desenvolvimento de Bioprocessos, Departamento de Engenharia Bioquímica, Escola de Química, Universidade Federal do Rio de Janeiro, Rio de Janeiro, Brazil; ^2^INRAE, Faculté des Sciences de Luminy, Aix Marseille Université, UMR 1163 Biodiversité et Biotechnologie Fongiques, Polytech Marseille, Marseille, France; ^3^Unidade de Espectrometria de Massas e Proteômica, Instituto de Bioquímica Médica Leopoldo de Meis, Universidade Federal do Rio de Janeiro, Rio de Janeiro, Brazil

**Keywords:** filamentous fungi, enzyme, LPMO, biomass, biofuels, biorefinery

## Abstract

The high demand for energy and the increase of the greenhouse effect propel the necessity to develop new technologies to efficiently deconstruct the lignocellulosic materials into sugars monomers. Sugarcane bagasse is a rich polysaccharide residue from sugar and alcohol industries. The thermophilic fungus *Myceliophthora thermophila* (syn. *Sporotrichum thermophilum*) is an interesting model to study the enzymatic degradation of biomass. The genome of *M. thermophila* encodes an extensive repertoire of cellulolytic enzymes including 23 lytic polysaccharide monooxygenases (LPMOs) from the Auxiliary Activity family 9 (AA9), which are known to oxidatively cleave the β-1,4 bonds and boost the cellulose conversion in a biorefinery context. To achieve a deeper understanding of the enzymatic capabilities of *M. thermophila* on sugarcane bagasse, we pretreated this lignocellulosic residue with different methods leading to solids with various cellulose/hemicellulose/lignin proportions and grew *M. thermophila* on these substrates. The secreted proteins were analyzed using proteomics taking advantage of two mass spectrometry methodologies. This approach unraveled the secretion of many CAZymes belonging to the Glycosyl Hydrolase (GH) and AA classes including several LPMOs that may contribute to the biomass degradation observed during fungal growth. Two AA9 LPMOs, called *Mt*LPMO9B and *Mt*LPMO9H, were selected from secretomic data and enzymatically characterized. Although *Mt*LPMO9B and *Mt*LPMO9H were both active on cellulose, they differed in terms of optimum temperatures and regioselectivity releasing either C1 or C1-C4 oxidized oligosaccharides, respectively. LPMO activities were also measured on sugarcane bagasse substrates with different levels of complexity. The boosting effect of these LPMOs on bagasse sugarcane saccharification by a *Trichoderma reesei* commercial cocktail was also observed. The partially delignified bagasse was the best substrate considering the oxidized oligosaccharides released and the acid treated bagasse was the best one in terms of saccharification boost.

## Introduction

The substitution of the fossil fuels is an important strategy to decrease the environmental impacts caused by the high levels of CO_2_ emission and greenhouse gases. The development of renewable energies is a promising strategy to reduce global warming damages and climate changes (Wyman, 2003; Farrell et al., 2006). Sugarcane is a well-established feedstock to produce first-generation ethanol (1G ethanol) (Smithers, 2014; Clauser et al., 2016). In 2019, more than 700 million tons were produced in the Brazilian territory. Although sugarcane as feedstock to 1G ethanol production is a successful model of the sugar-alcohol industry, its coproduct, sugarcane bagasse is still a challenging feedstock for the production of fuels and chemicals due to its recalcitrance to degradation (Eggert and Greaker, 2014; Khattab and Watanabe, 2019).

In nature, the most powerful natural decomposers of lignocellulosic biomass are filamentous fungi (wood-decaying fungi). They secrete a powerful group of enzymes called CAZymes^1^ (Lombard et al., 2014) that act synergistically to cleave the β1,4-β1,3 linkages of plant polysaccharides releasing as end products a range of monosaccharides that provide energy to the microorganisms intermingled in their environmental. *In vitro*, pretreatments of lignocellulosic biomass are required to render the substrate more accessible to the enzymes. Overall, additional cost reduction is desirable to achieve competitiveness of the second-generation (2G) ethanol production (Loqué et al., 2015).

Ten years ago, the discovery of a new class of copper-enzymes, the lytic polysaccharide monooxygenase (LPMO, EC 1.14.99.53-56), cleaving with an oxidative mechanism recalcitrant polysaccharides, revolutionized the concept of plant cell wall breakdown (Vaaje-Kolstad et al., 2010; Quinlan et al., 2011). These mono-copper enzymes are classified in Auxiliary Activity families AA9-AA11 and AA13-AA16 in the CAZy database and contain a characteristic histidine brace copper binding site. Due to their impressive boosting effect on the cellulose breakdown, LPMOs have rapidly become key components of commercial enzymatic cocktails (Johansen, 2016). Filamentous fungi, especially fungal saprotrophs, which degrade complex and recalcitrant plant polymers, are proficient secretors of LPMOs and their redox partners (Berrin et al., 2017). They secrete LPMOs that differ in substrate specificities, AA9 members target cellulose and hemicelluloses (Tandrup et al., 2018), AA11 LPMOs cleave chitin (Hemsworth et al., 2014), AA13 target starch (Vu et al., 2014b; Lo Leggio et al., 2015), AA14 recalcitrant xylans (Couturier et al., 2018) and AA16 cellulose (Filiatrault-Chastel et al., 2019). AA9 LPMOs occur either as isolated modules or appended to cellulose-binding modules (CBM1) at the C-terminal in agreement with the critical role of the N-terminal histidine for enzyme activity. The remarkable expansion of the AA9 family (more than 30 candidate proteins in some genomes) raises the question of its functional relevance at the organismal level. While all AA9 LPMOs characterized to date cleave cellulose, subtle differences have been observed in their regioselectivities acting either on the C1, C4 or C1 and C4 carbon of the glucose unit.

*Myceliophthora thermophila* syn. *Sporotrichum thermophilum* is a ubiquitous thermophilic fungus with a strong ability to hydrolyze all major polysaccharides found in biomass due to the secretion of many CAZymes (cellulases, xylanases, pectinases and some other miscellaneous enzymes) employed in various biotechnological applications (Karnaouri et al., 2014; Singh, 2016). Characterization of the biomass-hydrolyzing activity of wild and recombinant enzymes suggests that this mold is highly efficient in biomass decomposition at both moderate and high temperatures. The genome of *M. thermophila* encodes 23 AA9 LPMOs (Berka et al., 2011) of which nine have been characterized relative to their substrate specificity and/or regioselectivity (Vu et al., 2014a; Frommhagen et al., 2015, 2016, 2018; Karnaouri et al., 2017; Hangasky et al., 2018).

In this study, we investigated the enzymatic arsenal secreted by *M. thermophila* upon growth on sugarcane bagasse using proteomics. A large range of CAZymes targeting lignocellulose was observed in the secretomes and due to the importance of LPMOs, the activity of the two AA9 LPMOs was studied in different conditions to depict their contribution to sugarcane bagasse degradation.

## Materials and Methods

### Sugarcane Bagasse Substrates

The sugarcane bagasse (*Saccharum* spp.) was obtained from Boa Vista sugar-alcohol industry (Goiás, Br) and stored at room temperature. To use as substrate, the sugarcane bagasse not treated (SCBNT) was washed several times in water, dried at 45°C in an air oven for 3 days and milled (willed mill at Tecnal, TE-680, BR) in a sieve with 5 mm particle size. After, 1 kg was subjected to acid treatment using a 1.09% (v/v) H_2_SO_4_ solution with a solid-liquid proportion of 1:2.8 (w/v) for 30 min at 121°C (Betancur and Pereira, 2010). The acid pretreatment provided a liquid comprised mostly by hemicelluloses chemically hydrolyzed and a solid fraction composed mainly by cellulose and lignin (Khattab and Watanabe, 2019) that was separated by a pressing filtration at 10 kgf.cm^–2^. The solid phase was subsequently washed exhaustively until pH reached 5.0 and dried at 45°C in air oven for 3 days. The resulting feedstock is called the sugarcane bagasse acid treated (SCBAT). Subsequently, half of the SCBAT was submitted to alkaline pre-treatment conducted using sodium hydroxide 4% (w/v) at 121°C for 30 min with a solid-liquid-ratio of 1:20 (Vasquez et al., 2007). The acid and alkaline treated bagasse called sugarcane bagasse partially delignified (SCBPD) was washed several times until pH reached 5.0 and dried in an air oven at 45°C for 3 days.

### Sugarcane Bagasse Composition

The determination of the structural carbohydrates (cellulose and hemicellulose), lignin and ashes in SCBNT, SCBAT and SCBPD were derived through chemical hydrolysis with 72% (v/w) H_2_SO_4_ as described (Sluiter et al., 2008; Supplementary Table S1). The sugars released were measured by glucose oxidase assay (GOD) and 3–5, Dinitrosalycilic acid method (DNS) (Miller, 1959) using standard solution of glucose with the highest grade of purification to establish the sugars concentration. The hemicellulose portion was calculated subtracting DNS and glucose oxidase assay (GOD) values. All the analyses were carried out twice in triplicate on dried samples.

### Microorganism and Cultivation

The *Myceliophthora thermophila* (M7.7 equivalent to FGSC 26436) strain used in this study was maintained in glycerol 20% (v/v) in microtubes at 4°C. The suspension was inoculated onto fresh potato dextrose agar medium (PDA) plates and incubated at 50°C to grow for 4 days. Liquid culture media enriched with 15 g L^–1^ dry matter (d.m.) of either SCBAT or SCBPD were prepared as follow: 0.2 g L^–1^ yeast extract; 1.0 g L^–1^ peptone; 0.3 g L^–1^ urea; 0.4 g L^–1^ CaCl_2_.2H_2_O; 0.2 g L^–1^ MgSO_4_.7H_2_O; 2.0 g L^–1^ KH_2_PO_4_; and microelements solutions formed by 5.0 mg L^–1^ FeSO_4_.7H_2_O; 1.6 mg L^–1^ MnSO_4_.4H_2_O; 1.4 mg L^–1^ ZnSO_4_.7H_2_O, and 2.0 mg L^–1^ CuSO_4_.6H_2_O (modified from Mandels and Weber, 1969) and autoclaved. The ascospores were scraped from five plates with an inoculation loop and transferred to a 50 mL falcon tube containing 10 mL sterilized liquid culture medium. The spores were enumerated with a Malassez cell counting chamber under a microscope. Three culture media (pH 5.3) were inoculated with 106 spores mL^–1^. The fungal growth was carried out in 1 L flask with 100 mL of medium at 45°C for 2 days in an orbital shaker (New Brunswick Excella, São Paulo, Brazil). All experiments were performed in duplicate.

### Extract Production for Proteomic Analysis

The solid and liquid fractions were separated by a vacuum filtration using a glass wool (Merck, Rio de Janeiro, Brazil). The corresponding culture broths (secretomes) were first filtered using a 0.22 mm cut-off column and then concentrated using a 5 kDa cut-off column, both in a hollow fiber system (QuixStand, GE, Healthcare, São Paulo, Brazil). The resulted protein extract was further concentrated by precipitation or lyophilization. For protein precipitation the same volume of TCA/Acetone 20% v/v were added to 3 mL of samples and led at −20°C overnight. Supernatant was discarded after centrifugation at 12,000 *g* for 30 min and the precipitate was washed three times with 3 mL of cold acetone and centrifugated as previously described. Protein pellet were resuspended in 200 μL of sample buffer for electrophoresis (2% SDS and 0.5 M tris-HCl 10% glycerol) and 15 μL of each sample were loaded onto a 12.5% SDS-PAGE. In parallel, 15 mL of the corresponding protein concentrated were lyophilized (Freeze Dryer, LS6000, Terroni) resuspended in 1.0 mL citrate buffer pH 5.0, filtered in 0.22 μm pore size membrane (Syringe-driven Filters, Biofilm). After filtration, 20 μg of each sample were loaded onto a preparative SDS-PAGE gel 12% which was halted after 1 cm migration into the separation gel in order to obtain a single gel slice for each sample.

### Proteomic Analysis

Gel slices, from analytical and preparative SDS-PAGE were submitted to a tryptic digestion after a treatment with 5 mM DTT (dithiothreitol), followed by 15 mM iodoacetamide and trypsin in a 1:50 mass ratio to protein. The extracted peptides from the gel spots were analyzed by LC/MS-MS.

Samples from analytical SDS-PAGE, one slice each time, were loaded on a Waters Nano acquity system (Waters, Milford, MA, United States). The peptides were desalted on-line using a Waters Symmetry C18 180 μm × 20 mm, 5 μm trap column. The sample injection volume was typically 7.5 μL, and the LC was performed by using BEH 130 C18 100 μm × 100 mm, 1.7 μm column (Waters, Milford, MA) and eluting (0.5 μL min^–1^) with a linear gradient (10–40%) of acetonitrile containing 0.1% formic acid using 70 min running time. Samples were injected on-line into a Micro Q-Tof spectrometer (Waters, Milford, MA). The ESI voltage was set at 3500 V, the source temperature was 80°C and the cone voltage was 30 V. The instrument control and data acquisition were conducted by a MassLynx data system (Version 4.1, Waters), and experiments were performed by scanning from a mass-to-charge ratio (*m*/*z*) of 400–2000 using a scan time of 1 s, applied during the whole chromatographic process. The exact mass was determined automatically using the Q-Tof’s LockSpray^TM^ (Waters, Milford, MA). Data-dependent MS/MS acquisitions were performed on precursors with charge states of 2, 3 or 4 over a range of 50–2000 *m*/*z* and under a 2 *m*/*z* window. A maximum of three ions were selected for MS/MS from a single MS survey. Collision-induced dissociation (CID) MS/MS spectra were obtained using argon as the collision gas at a pressure of 40 psi, and the collision voltage was varied between 18 and 90 V depending on the mass and charge of the precursor.

The results from this method are referred to as 1-DE and data were processed using the ProteinLynx Global server (version 2.5, Waters). The processing automatically lock mass corrected the *m*/*z* scale of both the MS and MS/MS data utilizing the lock spray reference ion. Generated peak list files were submitted to Mascot-Matrix science search engine (Version 2.2) protein and peptide tolerance were set as 0.1 Da. All proteins with score enough to assure a *p*-value lower than 0.05 was considered as a positive identification.

Tryptic peptides, obtained from the preparative gel were loaded in quintuplicate on a Waters Nano acquity system (Waters, Milford, MA, United States). The peptides were desalted on-line using a Waters Symmetry C18 180 μm × 20 mm, 5 μm trap column. The sample injection volume was 2 μL according to previous injection used to normalize total ion intensity in all samples. Glycogen phosphorylase standard (Waters, Milford, MA, United States) was added to each sample so that 100 fmol was inject in each and all samples. The LC was performed by using a 1.7 μm HSS T3 130 C18 (150 μm × 75 mm) column (Waters, Milford, MA, United States) and eluting (0.5 μL/min) with a linear gradient (10–40%) of acetonitrile containing 0.1% formic acid using 150 min running time. Electrospray tandem mass spectra were obtained using a Waters SynaptTM G1 HD/MS High Definition Mass Spectrometer (Waters, Manchester, United Kingdom) interfaced to the Nano acquity system capillary chromatograph. The ESI voltage was set at 3500 V, the source temperature was 80°C and the cone voltage was 30 V. Instrument control and data acquisition were conducted by a MassLynx data system (Version 4.1, Waters), and experiments were performed by scanning from a mass-to-charge ratio (m/z) of 50–2000 using a scan time of 0.8 s.

The results from this method are referred to as label-free LC-MS/MS and data were processed using the Progenesis QI for proteomics version 2.0 software platform (Non-linear Dynamics, Waters, Manchester, United Kingdom), the exact masses was determined automatically using the Q-Tof’s LockSpray^TM^. For all identification analysis, raw data were search against *M. thermophila* Uniprot non-reviewed protein Database, including Human Keratin proteins and Sus scorfa Trypsin as possible contaminants. Cysteine carbamidomethylation was set as fixed modification, peptide N-terminal carbamidomethylation, methionine oxidation and asparagine deamidation were set as variable modification. Only peptide ions with PLGS score above 5.0 were considered as an identification match, and only proteins quantifications with ANOVA *p*-value below 0.05 were reported. Functional protein annotation was then performed using Uniprot and the CAZy database^1^.

### Cloning and Heterologous Expression of Two AA9 LPMOs in *Pichia pastoris*

The two target genes AA9 LPMOs, *Mt*LPMO9B (GenBank ID 11509292) and *Mt*LPMO9H (GenBank ID 11510592), in frame adding a C-terminal (His)_6_-tag to the recombinant proteins, were codon optimized for expression in *P. pastoris* Easy Select Expression System (Genewiz Inc., United States). The *Pme*I-linearized pPICZαA recombinant plasmids were inserted into *P. pastoris* competent cells by electroporation as previously described (Bennati-Granier et al., 2015; Haon et al., 2015). Zeocin-resistant transformants were then screened for protein production.

The best producing transformants were grown in 1 L of BMGY containing 1 mL L^–1^ of *Pichia* trace minerals 4 (PTM_4_) salts (2 g L^–1^ CuSO_4_.5H_2_O; 3 g L^–1^ MnSO_4_.H_2_O; 0.2 g L^–1^ Na_2_MoO_4_.2H_2_O; 0.02 g L^–1^ H_3_BO_3__;_ 0.5 g L^–1^ CaSO_4_.2H_2_O; 0.5 g L^–1^ CoCl_2_; 12.5 g L^–1^ ZnSO_4_.7H_2_O; 22 g L^–1^ FeSO_4_.7H_2_O; 0.08 g L^–1^ NaI; biotin and 1 mL L^–1^ concentrated H_2_SO_4_) in shaken flasks at 30°C in an orbital shaker (200 rpm) for 16 h to an OD600 of 2–6. Expression was induced by transferring the cells into 200 mL of BMMY containing 1 mL L^–1^ of PTM_4_ salts at 20°C in an orbital shaker (200 rpm) for another 3 days. Each day the medium was supplemented with 3% (v/v) methanol.

### Recombinants AA9 *Mt*LPMOs Purification

The supernatants were collected after harvesting cells by centrifugation at 3,500 *g* for 5 min at 4°C. After adjusting the pH to 7.8, the supernatants were filtered on 0.45 μm filters (Millipore, Molsheim, France) and loaded onto a 5 mL HisTrap HP columns (GE healthcare, Buc, France) connected to an Akta Xpress system (GE healthcare). Prior to loading, the columns were equilibrated with buffer A Tris-HCl 50 mM pH 7.8, NaCl 150 mM and imidazole 10 mM. The (His)6-tagged recombinant enzymes were eluted with buffer B Tris-HCl 50 mM pH 7.8, NaCl 150 mM and imidazole 500 mM. The fractions eluted containing the purified proteins were pooled, concentrated with a 10 kDa vivaspin concentrator unit (Sartorius, Plaiseau, France) and dialyzed against 50 mM sodium acetate buffer pH 5.2. The concentrated proteins were incubated with a double equimolar equivalent of CuSO_4_ in a cold room and buffer exchanged in 50 mM sodium acetate buffer pH 5.2 using extensive washing in a 10-kDa ultrafiltration to remove traces of CuSO_4_.

Protein concentrations were determined using a NanoDrop ND-2000 spectrophotometer (Thermo Fisher Scientific, IL, United States) by adsorption at 280 nm with theoretical molecular masses and molar extinction coefficients calculated from protein sequences using Expasy tools. An aliquot of 10 μg of each protein was loaded onto 10% Tris-glycine precast SDS-PAGE stain-free gel (Bio-rad, Marnes-la- Coquette, France) to check protein purity and integrity (Supplementary Figure S1). The molecular mass under denaturing conditions was determined with PageRuler Prestained Protein Ladder (Thermo Fisher Scientific).

### Activity of AA9 *Mt*LPMOs on Cellulosic Substrates

The activities of the AA9 *Mt*LPMOs were carried out by addition of 1 μM *Mt*LPMO9B or *Mt*LPMO9H in 50 mM sodium acetate pH 5.2, 1 mM of ascorbate as electron donor using 0.1% (w/v) phosphoric acid swollen cellulose (PASC) or Avicel as substrates. The cleavage assays occurred in a final reaction volume of 300 μL in a 2 mL tubes in a rotatory shaker at 850 rpm (Infors AG, Switzerland). PASC was prepared from Avicel as described by Wood (1988).

The enzymatic reactions were performed at 40°C and interrupted at 30 min, 1, 2, 6 and 24 h by boiling the samples for 10 min. The samples were then centrifuged at 10,000 *g* for 10 min and the supernatants were stocked in a cold room until analysis. To get insights about temperature dependence, the experiment was performed using PASC as substrate, in water baths adjusted for the desirable temperatures, all at the same time, from 30° to 80°C, except the room temperature experiment, which was done at 22°C on the bench. After 2 h, the samples were boiled for 10 min to stop the reaction and centrifuged as described above. The supernatants containing oligosaccharides and their corresponding aldonic acid and C4-gemdiol forms generated after PASC and Avicel cleavage were analyzed by high-performance anion exchange chromatography coupled with amperometric detection (HPAEC-PAD) as described by Westereng et al. (2013). Non-oxidized cello-oligosaccharides standards from DP2 to DP6 were purchased from Megazyme (Wicklow, Ireland) and C1-oxidized standards were produced from non-oxidized cello-oligosaccharides using a cellobiose dehydrogenase as described in Bennati-Granier et al. (2015). The experiments were performed in triplicates.

### Activities of AA9 *Mt*LPMOs on Sugarcane Bagasse and Saccharifcation Assays

The enzymatic reactions were performed in 2 mL tubes containing 20 g L^–1^ of either SCBAT or SCBPD in 50 mM sodium acetate pH 5.2, supplemented with 0.15 mg mL^–1^ of tetracycline as antibiotic, cycloheximide 0.04 mg mL^–1^ as antifungal agent, and 1 mM ascorbate. Reaction were performed at 30°C or 50°C in a rotatory shaker at 850 rpm and started by addition of 1 μM *Mt*LPMO9B or 1 μM *Mt*LPMO9H in 1 mL final volume in 2 mL tubes. After 16 h incubation, the samples were centrifuged at 5,000 *g* for 5 min, and aliquots of 0.25 mL from each sample were taken and boiled for 10 min, and oxidized products were analyzed on HPAEC-PAD. The experiments were performed in triplicates. The reminiscent 0.75 mL volume were used for saccharification assays. For this, 0.25 mL of *Trichoderma reesei* cocktail (Celluclast, Sigma, Reims, Fr) containing 0.7 filter paper Unit. G^–1^ d.m and 60 U.g^–1^ d.m of β-glucosidase (Novozymes 188) were added to the 2 mL tubes. The reaction was conducted for 4 h at 40°C and 850 rpm in a rotatory shaker (Infors AG, Switzerland). The glucose yield was measured by HPAEC-PAD. Control conditions were carried out without LPMO and without commercial *T. reesei* cocktail.

## Results

### Carbohydrate-Active Enzymes Secreted by *M. thermophila*

**FIGURE 1:**
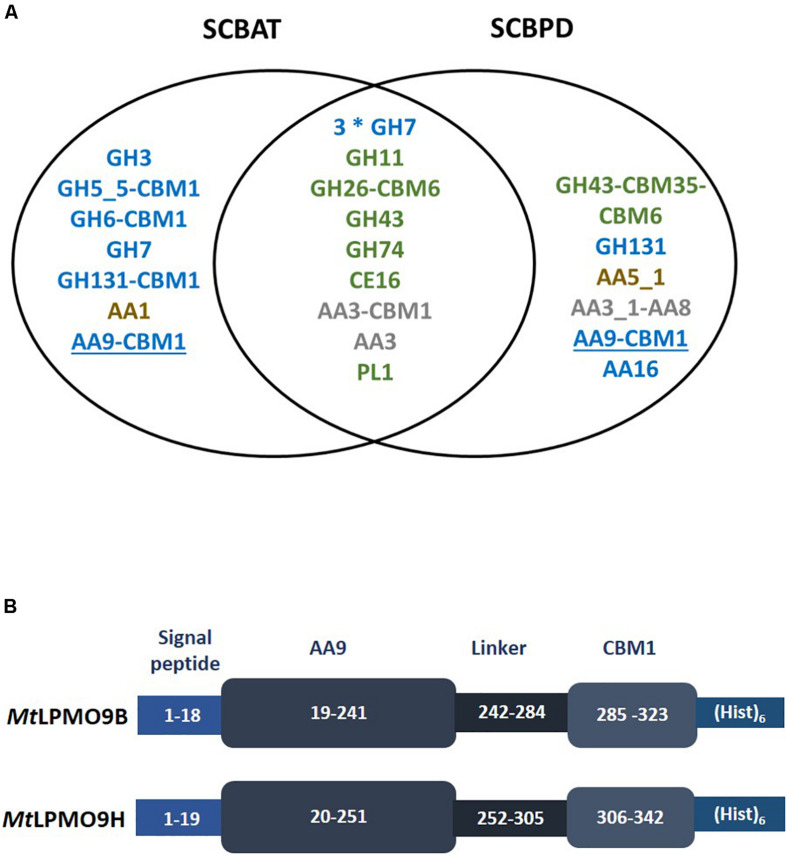
Proteomic analysis of *Myceliophthora thermophila* secretomes leading to the identification of two AA9 LPMOs. **(A)** List of CAZymes identified in the secretomes using proteomics. Only the CAZymes targeting plant cell wall polysaccharides are indicated. Putative cellulases in blue, hemicellulases and pectinases in green and lignin-active enzymes in brown. AA3 enzymes that may act in synergy with LPMOs are indicated in gray and AA9 LPMOs are underlined. **(B)** Schematic representation of the recombinant *Mt*LPMO9s expressed in this study. The numbers represent the amino-acid numbering in the primary structure of the polypeptide formed by signal peptide, AA9 catalytic domain, carbohydrate binding domain (CBM) and the C-terminal (His)6-tag.

The strain *M. thermophila* M7.7 was previously isolated from sugarcane bagasse piles (Moretti et al., 2012). We first pretreated sugarcane bagasse using different methods to induce the secretion of enzymes by *M. thermophila*. Composition analyses showed that both pretreated sugarcane bagasse samples, i.e., acid treated (SCBAT) and partially delignified (SCBPD), differed from the sugarcane bagasse not treated (SCBNT) in terms of cellulose, hemicellulose and lignin composition (Supplementary Table S1). The proteins secreted by *M. thermophila* upon growth on pretreated sugarcane bagasse feedstocks were collected at the initial stage of growth and analyzed by proteomics using two different methodologies (1-DE and label-free LC-MS/MS, see “Materials and Methods” section). Data analyzed using mass matching against predicted proteins inferred from genomic and transcriptomic sequence data of the *M. thermophila* ATCC 42464 (Berka et al., 2011) reported a total of 54 proteins, 21 identified by both methodologies, 16 only by label-free LC-MS/MS and 17 only by 1-DE as showed in Additional file (Supplementary Table S2). Out of the 54 proteins, 33 assigned as CAZymes (Figure 1A). Interestingly 10 of these CAZymes harbored at least one CBM module (from family CBM1, CBM6, CBM20, CBM35, CBM43) attached to their catalytic module. The analyses from the two methodologies used in this work showed that among the 54 proteins matched, 24 enzymes were found in both secretomes among which 15 CAZymes predicted to act on cellulose (GH7 cellobiohydrolase and GH131 endoglucanase) and hemicelluloses (GH11 endo-xylanase and GH26-CBM6 endo-mannanase). Of note, a family CE16 enzyme known to act on a wide range of carbohydrate acetyl esters (Puchart et al., 2016) could help the hemicellulases to deconstruct mannans and xylans. To complete this set of GHs, two AA3 oxidoreductases have been identified in both secretomes. Regarding the secretomes produced using SCBNT or SCBAT as inducer, several GHs were specifically identified in these secretomes among which a GH3 β-glucosidase and several endoglucanases among which a GH131 broad specificity endoglucanase bearing a CBM1 module (Lafond et al., 2012). One laccase (AA1) and one AA9 LPMO bearing a CBM1 were also identified only in the SCBAT secretome. Interestingly, the secretome produced on SCBPD displayed another AA9-CBM1 LPMO together with a LPMO from the AA16 family (Filiatrault-Chastel et al., 2019) and a cellobiose dehydrogenase (CDH) enzyme (AA3_1-AA8) that may serve as electron provider (Bey et al., 2013) for AA9 LPMOs.

### Sequence Analysis and Recombinant Production of Two *Mt*LPMO9s

The genes encoding two AA9 LPMOs were selected from the proteomic dataset. These two AA9 LPMOs were named according to previous work (Berka et al., 2011; Frommhagen et al., 2016; Karnaouri et al., 2017), i.e., *Mt*LPMO9B (GenBank ID AON76800.1) and *Mt*LPMO9H (GenBank ID AEO56542.1). The sequence of *Mt*LPMO9B is encoded by 323 amino-acids (aa), including the main catalytic domain (aa position at 19–241) appended to a carbohydrate binding module at the C-terminus (CBM1) (aa position at 285–323) and *Mt*LPMO9H is encoded by 342 aa including main catalytic domain (aa position at 20–251) carrying a CBM1 C-terminal extension (aa position at 306–342) (Figure 1B). In both enzymes, the AA9 catalytic module start with the canonical conserved histidine residue common to all LPMOs. Despite of the proximity in sequence length, sequence alignment of these AA9 LPMOs reported considerable variation with only 37% of identity (Supplementary Figure S2). It is indeed well known that in AA9 LPMOs, only the N-terminal part of the proteins are conserved, the rest of the sequence being highly variable (Lenfant et al., 2017). The recombinant production of these LPMOs was designed using their native signal sequence since it is important to ensure the correct processing of the signal peptide and therefore the correct binding of the catalytic copper ion to the histidine brace (Daly and Hearn, 2005; Ahmad et al., 2014; Ladevèze et al., 2017). Both enzymes were successfully expressed in *P. pastoris* and the best transformants reached a production yield above 100 mg per liter of culture. After purification, SDS-PAGE analysis revealed a unique band for each protein with apparent molecular weights higher than the ones expected from the sequences (Supplementary Figure S1) suggesting some glycosylations most probably in the linker regions.

### Activities of *Mt*LPMO9s on Cellulosic Substrates

**FIGURE 2:**
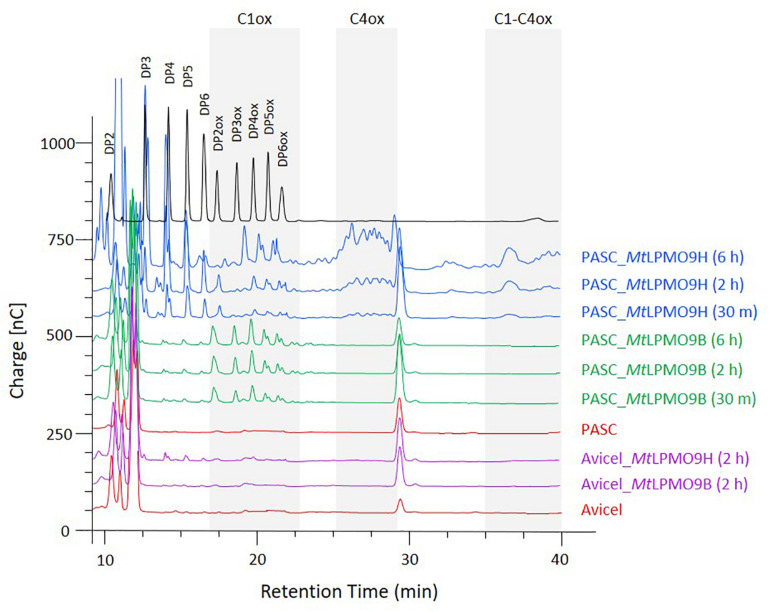
Time course analysis of the oligosaccharides released from PASC and Avicel by *Mt*LPMO9B and *Mt*LPMO9H. HPAEC chromatograms show the enzymes activity time dependence, in the presence of 1 mM Ascorbate. Standards are shown in black and control reactions in orange.

To analyze the oxidative cleavage of cellulosic substrates with different levels of recalcitrance, *Mt*LPMO9B and *Mt*LPMO9H were assayed on Avicel and PASC, using ascorbate as an electron donor and soluble products were analyzed using ionic chromatography (HPAEC-PAD). Overall, we observed that PASC was a better substrate than Avicel for both LPMOs (Figure 2). On PASC, *Mt*LPMO9B released small peaks of non-oxidized cello-oligosaccharides (DP2–DP6) and several peaks attributed to C1-oxidized products based on standards, mostly DP4ox followed by DP2ox (retention time between 17.5 and 22 min). Regarding *Mt*LPMO9H, a different pattern of soluble products was observed. We detected peaks corresponding to non-oxidized cello-oligosaccharides (DP2–DP6) and both C1- and C4-oxidized oligosaccharides. C1-oxidized products were mostly represented by DP4ox, followed by DP5ox and DP6ox and C4-oxidized products by species eluting between 26 and 29 min (Figure 2). *Mt*LPMO9H also released C1-C4 double oxidized products with late retention time between 35 and 40 min. These peaks increase significantly along the course of the reaction from 30 min to 6 h (Figure 2). The identification of C4-oxidized and C1-C4 oxidized products are based on previous analyses (Bennati-Granier et al., 2015; Ladevèze et al., 2017; Westereng et al., 2017). On Avicel, products were much less abundant and only significantly observed with *Mt*LPMO9H (Figure 2). From these data, it can be concluded that these two *Mt*LPMO9s display different regioselectivities and a preference for the amorphous cellulose.

### Effect of Temperature on *Mt*LPMOs Activity

**FIGURE 3:**
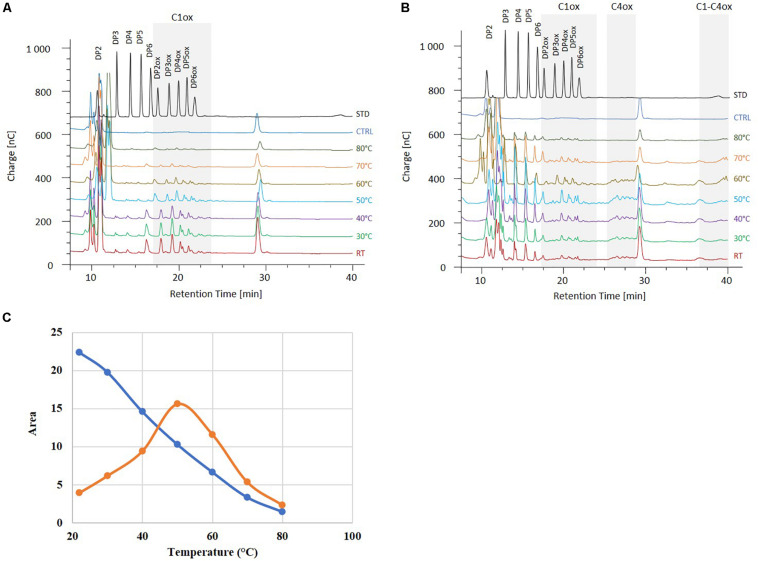
Effect of the temperature on the *Mt*LPMO9s activities. The oligosaccharides released by *Mt*LPMO9B **(A)** and 1 μM *Mt*LPMO9H **(B)** from PASC were analyzed by HPAEC after 2 h in the presence of 1 mM ascorbate. Standards (STD) and control reaction (CTRL) are indicated in black and blue, respectively. **(C)** Effect of the temperature on the activity of *Mt*LPMO9B (blue) and *Mt*LPMO9H (orange) represented by the release of C1-oxidized DP4 (DP4ox).

We further analyzed the activities of *Mt*LPMO9B and *Mt*LPMO9H in a biorefinery context. As *M. thermophila* is a thermophilic fungus, we hypothesized that *Mt*LPMO9s could present an interesting potential at elevated temperatures. Therefore, PASC cleavage was evaluated at different temperatures ranging from 22 to 80°C. From the chromatograms profile of the soluble non-oxidized and oxidized oligosaccharides produced, we observed that the *Mt*LPMO9s studied display distinct temperature preferences (Figure 3). The activity of *Mt*LPMO9B was maximum around 22–30°C and slowly decreased while temperature increased (Figure 3C). However, the activity of *Mt*LPMO9H displayed a typical bell shape with an optimum temperature around 50°C (Figure 3C). Although *Mt*LPMO9B and *Mt*LPMO9H showed optimal activity at 22–30°C and 50°C, respectively, both enzymes were able to release a broad range of cello-oligosaccharides with distinct intensity under a temperatures ranging from 22 to 70°C (Figures 3A,B). Interestingly, significant LPMO activity was still detected for both enzymes at 80°C (Figures 3A,B). These data illustrate the potential of these *Mt*LPMOs for biomass conversion at high temperatures.

### Contribution of *Mt*LPMO9s on Sugarcane Bagasse Saccharification

**FIGURE 4:**
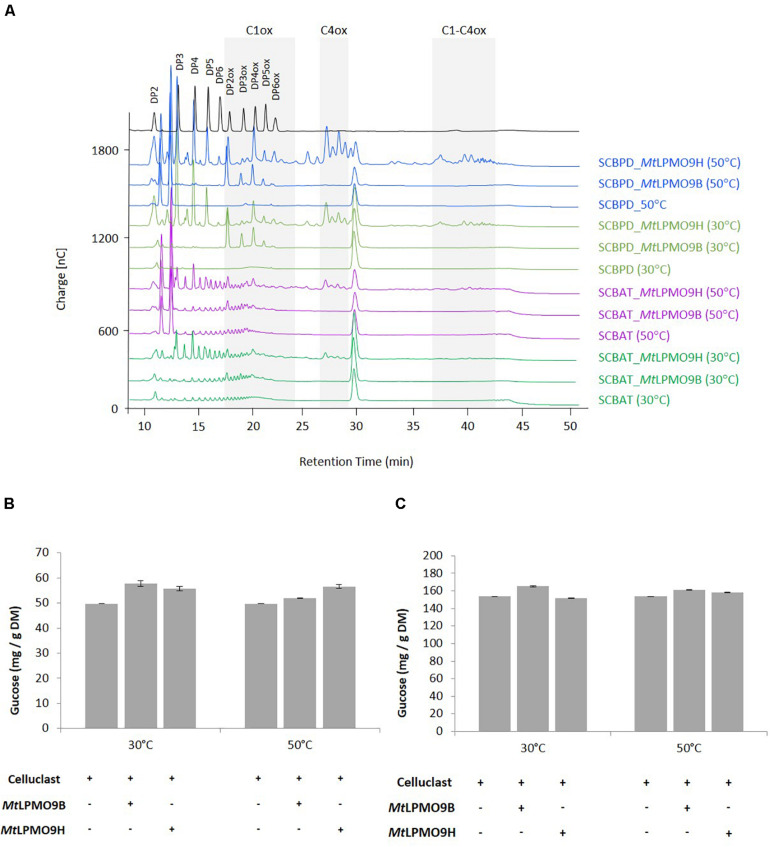
Contribution of *Mt*LPMO9s to the saccharification of pretreated bagasse. **(A)** Analysis of oligosaccharides released from sugarcane bagasse acid treated (SCBAT) and partially delignified (SCBPD) by *Mt*LPMO9B and *Mt*LPMO9H at 30 and 50°C for 16 h before the addition of the *T. reesei* cocktail. All assays were run in the presence of 1mM ascorbate as electron donor. **(B,C)** Saccharification yield of the sugarcane bagasse **(B)** acid treated (SCBAT) and **(C)** acid/alkaline treated (SCBPD). The glucose released was quantified by HPAEC. Errors bars indicate standard deviations from triplicate samples.

To investigate the activities of *Mt*LPMO9s on biomass, both enzymes were tested on SCBAT and SCBPD pretreated bagasse feedstocks at different temperatures in a sequential reaction with *Mt*LPMO9s first before addition of a commercial *T. reesei* cocktail (see section “Materials and Methods”). Before addition of the *T. reesei* cocktail, we analyzed the action of *Mt*LPMO9s to make sure they were able to access and cleave cellulose present in pretreated bagasse feedstocks. The HPAEC-PAD chromatograms revealed the release of oxidized products from SCBAT and SCBPD by each of the *Mt*LPMO9s tested alone (Figure 4A). As expected, from the SCBPD substrate, *Mt*LPMO9B releases C1-oxidized cello-oligosaccharides, mainly from DP2ox to DP6ox while *Mt*LPMO9H releases a mixture of C1, C4, and C1/C4 oxidized products (Figure 4A). When assayed on SCBAT, a biomass with a lower content of cellulose and a higher level of recalcitrance than SCBPD, the *Mt*LPMO9 enzymes were still able to access cellulose and release a range of oxidized cello-oligosaccharides (Figure 4A). The strongest effect in term of soluble products released (both oxidized and non-oxidized) was observed using *Mt*LPMO9H at 50°C on SCBPD (Figure 4A). After addition of the commercial *T. reesei* cellulase cocktail, we observed a significant improvement of the glucose yield (up to 16% improvement) for each *Mt*LPMO9s with both SCBAT and SCBPD bagasse samples (Figures 4B,C). The most striking effects were observed at the optimal temperature of each enzyme. Overall, the partially delignified bagasse (SCBPD) was the best substrate considering the oxidized oligosaccharides released and the acid treated bagasse (SCBAT) was the best one in terms of saccharification boost. However, it should be noted that the SCBPD pretreatment was much more efficient as it allowed the release of three times higher yield of glucose compared to the SCBAT pretreatment (Figures 4B,C).

## Discussion

In this study, we focused on the enzymatic capabilities of *M. thermophila* on sugarcane bagasse, which is a relevant 2G feedstock in Brazil (Betancur and Pereira, 2010). After pretreatment of bagasse with different methods leading to feedstocks with various cellulose/hemicellulose/lignin proportions, we analyzed the enzymes secreted by this fungus at an early stage of bagasse degradation. This approach unraveled the secretion of several AA enzymes, including AA9 LPMOs that may act in synergy with cellulases. This early stage secretion of LPMO enzymes was also observed in the fungus *Laetisaria arvalis*, which is highly efficient on cellulose and known to secrete LPMOs before cellulases (Navarro et al., 2014). Another interesting observation is that the two AA9 LPMOs identified in the secretomes both display a CBM1 module. The preferential secretion of CBM-containing LPMOs by filamentous fungi is a trend already observed in *Podospora anserina* (Poidevin et al., 2014) and other fungal saprotrophs (Berrin et al., 2017) and could indicate a preferential role of these enzymes in lignocellulose degradation. Recent studies showed the functional relevance of CBM1 modules to promote LPMO binding and improve activity (Courtade et al., 2018; Chalak et al., 2019). For all these reasons, we decided to further study these AA9 LPMOs identified in *M. thermophila* secretomes. The *Mt*LPMO9B was previously studied to assess AA9 LPMO electron donor dependence (Frommhagen et al., 2016) and the *Mt*LPMO9H was studied in synergism with the GH5 endoglucanase from *M. thermophila* on PASC and pretreated wheat straw (Karnaouri et al., 2017). We comparatively characterized these *Mt*LPMO9s to identify interesting properties for the degradation of sugarcane bagasse. Our comparative assays using cellulose as model substrate revealed differences in regioselectivity and in optimum temperature cleavage. As compared to *Mt*LPMO9B, which was only active on amorphous cellulose, *Mt*LPMO9H was able to target both amorphous cellulose and crystalline cellulose. In terms of stability to temperature, *Mt*LPMO9H displayed a much better thermostability compared to *Mt*LPMO9B with an optimum temperature of 50°C but interestingly some activity was still detected for both enzymes at elevated temperatures. This observation confirms the strong binding capacity of copper to the histidine brace of LPMOs, even at elevated temperature. A strong binding of these LPMOs to cellulose could also protect the enzyme from auto-inactivation as demonstrate by Kracher et al. (2018). Indeed, a stabilizing effect of the substrate was demonstrated on the apparent transition midpoint temperature of the reduced, catalytically active LPMO enzyme. This stability at elevated temperature is of great interest for saccharification of biomass. In our study, both LPMOs degrade pretreated sugarcane bagasse composed by distinct cellulose and lignin ratio. The pretreatment methods used in this study significantly affected the overall glucose yield but also the boosting effect observed for each LPMO suggesting that multiple factors are playing a role in this process. The regioselectivity of each LPMO and synergy with the cellobiohydrolases present in the *T. reesei* cocktail could be of importance as well as some inhibitors embedded in the sugarcane bagasse after pretreatment. Moreover, although it was not the focus of the present study, inhibitors molecules released during pretreatment can also affect the final desired product yield during the fermentation step. Another important criterion to consider when the objective is to maximize saccharification is to find the good balance between LPMO oxidation vs. cellulases hydrolysis. Indeed, too much oxidation at the surface of cellulose might not benefit the overall cellulose turnover to glucose. This could explain the reason why the partially delignified bagasse was the best substrate when considering the oxidized oligosaccharides released after LPMO action and the acid treated bagasse, which was the best one in terms of saccharification boost.

## Data Availability Statement

All datasets presented in this study are included in the article/Supplementary Material.

## Author Contributions

MG, NP, and J-GB conceived the study. MG, RZ, NP, and J-GB planned the experiments. MG, MH, SG, AO-C, and AM carried out the experiments. MG produced the secretomes. AO-C and AM carried out the proteomic analyses. MG and MH produced recombinant proteins. MG and SG performed substrate degradation assays and HPAEC–PAD analyses. MG, MH, SG, RZ, NP, and J-GB contributed to the interpretation of the results. NP and J-GB supervised the work. MG and J-GB wrote the manuscript. All authors read and approved the final version of the manuscript.

## Conflict of Interest

The authors declare that the research was conducted in the absence of any commercial or financial relationships that could be construed as a potential conflict of interest.

## Footnotes

1 http://www.cazy.org

## Supplementary Material

TABLE S1 Compositional analysis of sugarcane bagasses used in this study.

TABLE S2 Proteins identified in the *M. thermophila* secretomes using proteomics.

FIGURE S1 SDS PAGE of purified recombinant *Mt*LPMO9B (1-2) and *Mt*LPMO9H (3-4). A total of 5 μg (1 and 3) and 10 μg (2 and 4) of purified enzyme were loaded onto the gel. MW standards (kDa) and indicated in (5).

FIGURE S2 Structure-based sequence alignment of the *Mt*LPMO9s studied. The conserved residues are shown in dark blue and the residues with similar physico-chemical properties are indicated in red. The residues forming the histidine brace are shown in light blue. The loops regions and disulfide bridges are also indicated. The sequence numbering start at the N-terminal Histidine (H1) and neither the linker nor the CBM are shown. The alignment was made using EMBL-EBI services and Pairwise Sequence Alignment (PSA).
