# Supplementary material for: Evaluation of the Enzymatic Arsenal Secreted by Myceliophthora thermophila During Growth on Sugarcane Bagasse With a Focus on LPMOs
Source: Front Bioeng Biotechnol. 2020 Aug 25;8:1028. doi: 10.3389/fbioe.2020.01028 (PMC7477043; doi:10.3389/fbioe.2020.01028)

Table S1: Biomass composition of the feedstock used in this study Sugarcane bagasse not treated (SCBNT), acid treated (SCBAT) and partially delignified (SCBPD).

	Cellulose (%)	Hemicellulose (%)	Lignin (%)
SCBNT	33	29	23
SCBAT	40	12	27
SCBPD	70	14	7.6

Table S2: Proteins identified in the *M. thermophila* secretomes using proteomics.

Gene	Functional annotation	CAZy (sub)family	Methodologies				Synapt Differential expression		
			1-DE		Label free -LC-MS/MS		Av. Norm. Abundance		LOG2 Fold Change
			SCBAT	SCBPD	SCBAT	SCBPD	SCBAT	SCBPD	SCBAT/SCBPD
MYCTH_66804	β-glucosidase	GH3	x				NI		
MYCTH_86753	endo-1,4-beta-glucanase	CBM1-GH5_5	x		x		378	138	1,5
MYCTH_66729	β-cellobiohydrolase	CBM1-GH6	x		x		1104	57	4,3
MYCTH_97137	β-cellobiohydrolase	GH7	x	x	x	x	14234	7139	1,0
MYCTH_116157	endo-1,4-beta-glucanase	GH7	x	x	x	x	347	279	0,3
MYCTH_109566	β-cellobiohydrolase	GH7	x	x	x	x	9586	493	4,3
MYCTH_111372	endo-1,4-beta-glucanase	GH7	x				NI		
MYCTH_99786	endo-1,4-beta-xylanase	GH11			x	x	441	49	3,2
MYCTH_72393	glucoamylase	GH15-CBM20	x				NI		
MYCTH_2315557	endo-1,3-beta-glucanase	GH16 (GPI-anchored)	x		x	x	484	894	-0,9
MYCTH_2315007	lichenase	GH17 (GPI-anchored)				x	302	743	-1,3
MYCTH_99077	β-mananase	GH26_CBM6	x	x			NI		
MYCTH_50820	arabinofuranosidase	GH43-CBM35-CBM6				x	162	442	-1,4
MYCTH_2072383	arabinofuranosidase	GH43	x		x		202	8	4,6
MYCTH_2305407	β 1,3 glucanase	GH55	x	x		x	714	668	0,1
MYCTH_102522	exo-1,3-β-glucosidase	GH55	x	x			NI		
MYCTH_66349	1,3 β glucanosyltranferase	GH72 (GPI-anchored)		x	x	x	3174	6430	-1,0
MYCTH_106819	1,3 β glucanosiltranferase	GH72-CBM43 (GPI-anchored)				x	217	1038	-2,3
MYCTH_71748	1,3 β-glucanosyltransferase	GH72 (GPI-anchored)				x	32	252	-3,0
MYCTH_116384	xyloglucanase	GH74	x	x	x	x	1345	1593	-0,2
MYCTH_2307666	exo-α-1,6-mannosidase	GH125	x				NI		
MYCTH_2295704	glucanase	GH131			x	x	264	805	-1,6
MYCTH_112399	glucanase	GH131-CBM1	x				NI		
MYCTH_84133	acetylesterase	CE16	x	x			NI		
MYCTH_75296	multicopper oxidase	AA1	x				NI		
MYCTH_111388	GMC oxidoreductase	AA3-CBM1	x	x		x	1479	426	1,8
MYCTH_2299749	GMC oxidoreductase	AA3	x	x	x	x	378	1912	-2,3
MYCTH_2294895	glyoxal oxidase	AA5_1		x		x	123	439	-1,8
MYCTH_81925	cellobiose dehydrogenase	AA8-AA3_1		x		x	174	1988	-3,5
MYCTH_80312	LPMO	AA9-CBM1			x		404	74	2,4
MYCTH_46583	LPMO	AA9-CBM1	x				NI		
MYCTH_2311254	LPMO	AA16				x	50	489	-3,3
MYCTH_52463	pectin lyase	PL1	x	x	x	x	1549	1178	0,4
MYCTH_33936	No significant hit		x	x	x		1885	80	4,6
MYCTH_59005	No significant hit					x	310	13852	-5,5
MYCTH_103702	aldose epimerase		x	x	x	x	7580	17895	-1,2
MYCTH_2298860	No significant hit		x	x	x	x	6335	7659	-0,3
MYCTH_2299608	No significant hit					x	2651	483	2,5
MYCTH_2299856	No significant hit		x		x		349	60	2,5
MYCTH_2307339	No significant hit		x	x		x	224	813	-1,9
MYCTH_2078150	No significant hit		x	x		x	165	1859	-3,5
MYCTH_75329	No significant hit					x	97	512	-2,4
MYCTH_2301054	endopeptidase					x	129	748	-2,5
MYCTH_2058204	No significant hit				x	x	208	40	2,4
MYCTH_2311417	No significant hit				x	x	20	2	3,2
MYCTH_110821	No significant hit					x	3	189	-6,0
MYCTH_2306748	No significant hit					x	7	118	-4,1
MYCTH_2299879	No significant hit		x	x			NI		
MYCTH_2310837	No significant hit		x	x			NI		
MYCTH_2310464	No significant hit		x				NI		
MYCTH_80916	catalase		x				NI		
MYCTH_112714	No significant hit		x				NI		
MYCTH_2308567	No significant hit		x				NI		
MYCTH_2312740	No significant hit		x				NI		

Figure S1: Structure-based sequence alignment of the *Mt*LPMO9s studied. The conserved residues are shown in dark blue and the residues with similar physico-chemical properties are indicated in red. The residues forming the histidine brace are shown in light blue. The loops regions and disulfide bridges are also indicated. The sequence numbering start at Histidine N-terminal (H1) and neither the linker nor the CBM are shown. The alignment was made using EMBL-EBI services and Pairwise Sequence Alignment (PSA).

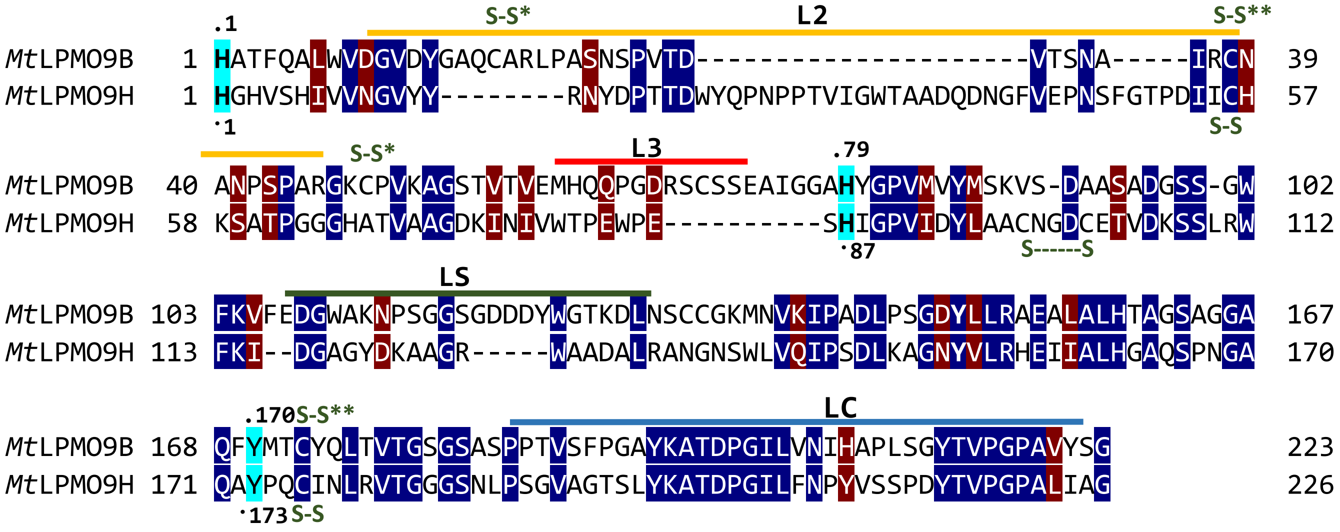

Figure S2. SDS PAGE of purified recombinant *Mt*LPMO9B (1-2) and *Mt*LPMO9H (3-4). A total of 5 µg (1 and 3) and 10 µg (2 and 4) of purified enzyme were loaded onto the gel. MW standards (kDa) and indicated in (5).

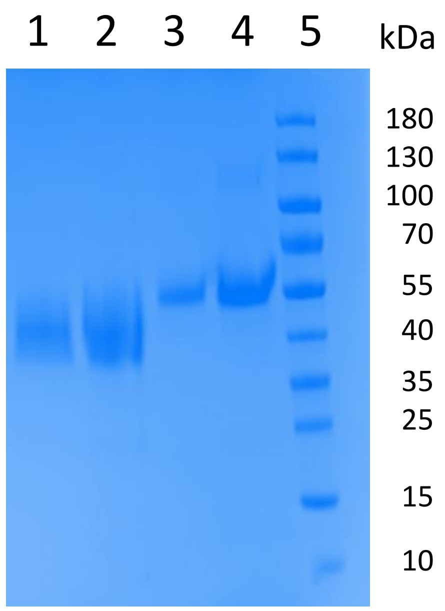

Supplement: TABLE S1 — Compositional analysis of sugarcane bagasses used in this study. [file Data_Sheet_1.PDF]
